# Leveraging Quadratic Polynomials in Python for Advanced Data Analysis

**DOI:** 10.12688/f1000research.149391.1

**Published:** 2024-05-17

**Authors:** Rostyslav Sipakov, Olena Voloshkina, Anastasiia Kovalova

**Affiliations:** 1 Department of Environmental Protection and Occupational Safety Technologies, Kyiv National University of Construction and Architecture, Kyiv, 03037, Ukraine

**Keywords:** python, quadratic polynomials, analyzing data, polynomial model

## Abstract

**Objectives:**

This study aims to provide a comprehensive overview of the role of quadratic polynomials in data modeling and analysis, particularly in representing the curvature of natural phenomena.

**Methods:**

We begin with a fundamental explanation of quadratic polynomials and describe their general forms and theoretical significance. We then explored the application of these polynomials in regression analysis, detailing the process of fitting quadratic models to the data using Python libraries NumPy and Matplotlib. The methodology also included calculation of the coefficient of determination (R-squared) to evaluate the polynomial model fit. This study utilizes illustratively generated data to demonstrate the application of quadratic polynomials in Python for robust data analysis.

**Results:**

Using practical examples accompanied by Python scripts, this study demonstrated the application of quadratic polynomials to analyze data patterns. These examples illustrate the utility of quadratic models in applied analytics.

**Conclusions:**

This study bridges the gap between theoretical mathematical concepts and practical data analysis, thereby enhancing the understanding and interpretation of the data patterns. Furthermore, its implementation in Python, released under MIT’s license, offers an accessible tool for public use.

## Plain Language Summary

This study examines how quadratic polynomials, which are mathematical equations used to model and understand patterns in data, can be effectively applied using Python, a versatile programming language with libraries suited for mathematical and visual analysis. Researchers have focused on the adaptability of these polynomials in various fields, from software analytics to materials science, in order to provide practical Python code examples. They also discussed the predictive accuracy of the method, confirmed through a statistical measure called R-squared, and acknowledged the need for future research to integrate more complex models for richer data interpretation.

## 1. Introduction

In our exploration of the quadratic polynomials in Python for data analysis, we found significant contributions across various domains, exemplifying their utility and versatility. Gong and Zhang (2021) presented a compelling application for predicting Python usage trends, and demonstrated a robust model fit with practical implications for software analytics. Python provides an ideal environment for the rapid prototyping of data analytic tools and includes powerful tools for visualization, data sharing, and statistical analysis, such as Matplotlib, iPython, NumPy, and SciPy ( Alexander et al., 2017).

This underscores the ability of quadratic polynomials to capture complex patterns beyond the realms of traditional linear models. In the context of quadratic polynomial regression, it is essential to note that quadratic polynomial step regression is an advanced tool capable of utilizing orthogonal experimental data to build a regression model, while avoiding instability in the regression coefficients owing to the multicollinearity of the variables ( Wang et al., 2014). This highlights the potential of quadratic polynomials for handling complex data relationships and providing accurate regression models. Gong and Zhang (2021) developed a polynomial regression model to predict the Python usage trends. Their model, which demonstrated high accuracy with a training set score of 0.912862 and a test set score of 0.886600, highlighted the effectiveness of quadratic polynomials in forecasting software usage patterns ( Gong and Zhang, 2021) *.*

Aladesanmi et al. (2021) illustrated the adaptability of quadratic polynomials in material science was illustrated by Aladesanmi et al. (2021) *,* who applied these models to understand the wear rate and hardness of nanocomposites. Aladesanmi et al. (2021) employed quadratic polynomial regression to analyze the relationship between material hardness and wear rate in Ti and TiB2 nanocomposites. Their findings, indicating a better fit for the quadratic model with an Adjusted R-squared value of 0.8883, underscores the utility of quadratic polynomials in material science research ( Aladesanmi et al., 2021). In epidemiology, Yadav (2020) leveraged quadratic polynomial regression models to analyze the COVID-19 epidemic in India, demonstrating its effectiveness in epidemic forecasting. This example reflects the predictive power of mathematical models and their crucial role in public health planning and responses ( Yadav, 2020 *).* In the context of urban development and assessment of geotechnical conditions, the incorporation of Python for data analysis, particularly through quadratic polynomials, can significantly enhance the understanding and monitoring of complex ground conditions ( Kaliukh et al., 2022).

In summary, Python, with its extensive libraries and capabilities for rapid prototyping, visualization, and scientific computation, provides a robust platform for leveraging quadratic polynomials in advanced data-analysis tasks.

## 2. Methods

### 2.1 Design and development environment

In this study, we focused on applying quadratic polynomials in Python for data analysis, highlighting the importance of these mathematical expressions in modeling and interpreting complex datasets using the following key concepts:

– Quadratic polynomials: Defined by the general form a $x^{2}+\mathrm{bx}+c$ , where (a),(b),(c) , are coefficients. These polynomials are essential for capturing curvature in datasets indicative of various natural and human-made phenomena.
– Python libraries: NumPy is open source and is available at https://numpy.org, were used for numerical computations, and Matplotlib also is open source and is available at https://matplotlib.org), was used to plot the data and polynomial curves, showing how these tools were integrated for data analysis.
– Regression analysis: Explains how quadratic polynomials can be fitted to data points to model relationships within the data, emphasizing practical applications through Python coding examples.
– Coefficient of determination (R-squared): Discuss the computation and interpretation of R-squared to measure how well the polynomial model fits the data.

A quadratic polynomial is an algebraic equation of the second degree, which includes a term raised to a power of two (squared). The general form of a quadratic polynomial is $\;y=ax^{2}+\mathrm{bx}+c$ , where (y) is the dependent variable; (x) is the independent variable; and (a),(b),(c) are the coefficients of the polynomial estimated by the regression model. The quadratic term ( $ax^{2})$ allows the model to capture the curvature in the data, which is indicative of acceleration increases or decreases that are common in many natural phenomena.

Some key features of quadratic polynomials are that they have two terms with a variable (x) - one is (x) - squared, and the other is (x) to the first power. The $\left(x^{2}\right)$ term has a non-zero coefficient (a) . This makes it a quadratic polynomial rather than a linear polynomial. When plotted, quadratic polynomials form a parabolic shape rather than a straight line. The quadratic polynomials have up to two distinct real roots for the equation $x^{2}+\mathrm{bx}+c=0$ . These solutions were obtained by factoring or by using a quadratic formula. Examples of quadratic polynomials include the vertex form $y=a{\left(x-h\right)}^{2}+k$ , and the standard form $y=ax^{2}+\mathrm{bx}+c$ . A quadratic polynomial has a squared, linear, and constant term, graphs as a parabola, and two roots at most.

Understanding their structures allows many mathematical and real-world problems to be solved. We provide an example of the Python script below, which employs a quadratic polynomial fitting technique–a method used in regression analysis to model the relationship between a dependent variable and one or more independent variables. In this case, the independent variable is time (represented in months) and the dependent variable is the metric of interest (such as pollution levels and sales figures).

After fitting the quadratic polynomial to the data, the script generated a smooth fitted curve that represented the estimated values of the dependent variable across a range of independent variables. This curve helps to visualize the overall trend and any potential seasonal patterns or anomalies in the dataset.

The coefficient of determination, commonly known as R-squared $\left(R^{2}\right)$ , was then calculated to quantify the goodness of fit of the polynomial model. It is a statistical measure that indicates the proportion of variance in the dependent variable that is predictable from independent variable(s). An $\left(R^{2}\right)$ value of one (1) indicated a perfect fit, indicating that the model explained all the data variability around its mean. In contrast, an $\left(R^{2}\right)$ value closer to zero (0) indicates that the model fails to accurately model the data.

For more in-depth information on quadratic polynomial fitting and calculation of the coefficient of determination, the following sources ( Norman R. Draper and Harry Smith, 2014; Douglas et al., 2021) provide a comprehensive overview of seminal works on regression analysis and detailed explanations of various regression techniques, including quadratic polynomial fitting and interpretation $\left(R^{2}\right)$ .

Next, we applied quadratic polynomial fitting and R-squared in Python for the data analysis. In this case, the Python script exemplifies the application of regression analysis using the NumPy and Matplotlib libraries to model and visualize trends in time-series data. A core component of this analysis is the fitting of a quadratic polynomial to the data, grounded in the principles of statistical learning.

In Python, this was achieved using the 'Polynomial.fit' method from the NumPy library, which computes the least-squares fit of a polynomial of specified degree to the given data. The snippet calculates the optimal values for coefficients (a),(b),(c) that minimize the sum of the squared differences between the observed values and values predicted by the polynomial, thereby effectively “fitting” the curve to the data, which Python-formatted version of code snippet similar to:

*# Fit the quadratic polynomial*
coefs = Polynomial.fit(months, values, 2).convert().coef


With the fitted polynomial, our script generates a curve across a continuum of points within the data range, which was visualized using Matplotlib’s plotting function and the Python-formatted version of the code snippet similar to:

*# Generate a smooth curve by evaluating the polynomial at many points*
x = np.linspace(months.min(), months.max(), 200)
y = coefs[0] + coefs[1] * x + coefs[2] * x**2

*# Plot the data and the fitted curve*
plt.plot(x, y, color='purple', label='Fitted curve')


The coefficient of determination, $R^{2}$ , was subsequently computed to assess the fit quality. Python was used to compare the variance of the residuals (the differences between the observed and predicted values) with the total variance of the data, and the corresponding code snippet is similar to:

*# Calculate R-squared value*
residuals = values - coefs[0] + coefs[1] * months + coefs[2] * months**2
ss_res = np.sum(residuals**2)
ss_tot = np.sum((values - np.mean(values))**2)
r_squared = 1 - (ss_res / ss_tot)


An $\left(R^{2}\right)$ value close to one (1) suggests that the model explains a large portion of the variance in the response variable, indicating a strong fit. Conversely, a value near zero (0) suggests the model does not explain the variance well.

The following sources ( VanderPlas, 2016; McKinney, 2017) provide a comprehensive overview of Python’s theoretical background and practical application. These resources offer a deep dive into data analysis using Python, including comprehensive guidance on regression analysis, and robust examples that bridge theory with practice.

### 2.2 Implementation

This section details the implementation of quadratic polynomial models in Python that are used in various applications, as demonstrated in this study. The core of the implementation involved the use of Python NumPy and Matplotlib libraries for mathematical operations and visualizations. The polynomial model is defined by the equation ${ax}^{2}+\mathrm{bx}+c$ , where (a),(b),(c) , are the coefficients optimized to fit the data points collected in different studies. The fitting process utilizes the 'Polynomial.fit' method, which employs a least-squares polynomial fit. To ensure robustness and accuracy, the implementation also included calculation of the coefficient of determination $\left(R^{2}\right)$ using NumPy’s correlation function. This metric helps to assess the polynomial fit to the data, which is essential for the applications discussed, ranging from trend analysis in software usage to predicting the material properties of nanocomposites.

In the next step, we used Python to present an applied exploration of quadratic polynomial fitting and the coefficient of determination (R-squared) within the context of data analysis. The following Python script is a practical implementation tool for researchers and analysts: It begins by prompting the user to describe the dataset, such as a location or a specific environmental metric, such as the PM2.5 air pollution index. This interactivity ensures that the resulting visualization is tailored and informative. The Python-formatted version of the code snippet of this part of our script similar to:

*# User inputs for the descriptive elements of the plot*
description = input("Enter the location description (e.g., Kyiv, Shcherbakovskaya St.):")
pollution_name = input("Enter the pollution name (e.g., PM2.5):")
y_label = input("Enter the y-axis label (e.g., PM2.5 Index):")


The script reads data from a CSV file using Pandas, a library that excels in data manipulation. The data consists of monthly observations of the chosen metric. You can see this implementation in the Python-formatted version of the code snippet similar to:

*# Read data from a CSV file*
*# Use the direct link to the raw CSV file from the GitHub repository*
data = pd.read_csv('https://raw.githubusercontent.com/rsipakov/QuadraticPolynomialsPyDA/main/notebooks/pm_data.csv')
*# Or downloading CSV file to the local*
*# data = pd.read_csv('/path/pm_data.csv') # Update the path to your CSV file*
months = data['Month'].to_numpy()
values = data['Values'].to_numpy()


As described above, with the data in hand, the NumPy library’s 'Polynomial.fit' function is employed to fit a quadratic polynomial to these observations. This is an essential step in modeling nonlinear behavior, accommodating potential fluctuations in data that a simple linear model would miss. Subsequently, the script computes the fitted values and leverages them to calculate the R-squared values. This statistic conveys the proportion of variance in the dependent variable explained by the independent variable. The Matplotlib library was then used to graphically represent the data along with the fitted curve, visually comparing the actual data points with those of the predictive model.

After developing the script using the quadratic polynomial models described above, the complete Python code was hosted on GitHub ( Sipakov, 2024), enabling replication and further exploration of the findings. To facilitate ease of use and accessibility, the code was made available through MyBinder.org ( https://mybinder.org/v2/gh/rsipakov/QuadraticPolynomialsPyDA/main), allowing it to operate in a live environment without the need for local setup. This implementation ensures that other researchers can directly interact with the codebase, providing a dynamic way to validate and extend research findings.

### 2.3 Operation

The software tool based on quadratic polynomial models requires the following system setup and workflow: Operating System—Windows, macOS, or Linux; Python Version—Python 3.6 or later; dependencies —NumPy, Matplotlib (the latest versions are recommended), memory, at least 4GB of RAM; Processor, minimum 1GHz processor, or faster. The software is accessible through MyBinder.org, requires no local installation, and is fully configured to run in any web browser, ensuring its ease of use and reproducibility.

### 2.4 Installation process

To begin the installation process, it is imperative to ensure that Python is installed in the operating system. If Python is not present, it can be acquired from Python’s official website python.org. After successful installation of Python, the next step involved installing the necessary libraries. This can be achieved through the Python Package Index (PyPI) using a PIP installer. Execute the following command in the command prompt or terminal to install the required libraries: 'pip install numpy matplotlib'. This command installs NumPy, which is essential for numerical computations, and Matplotlib, a library for plotting graphs and effectively visualizing the data.

## 3. Results

**Figure 1.**
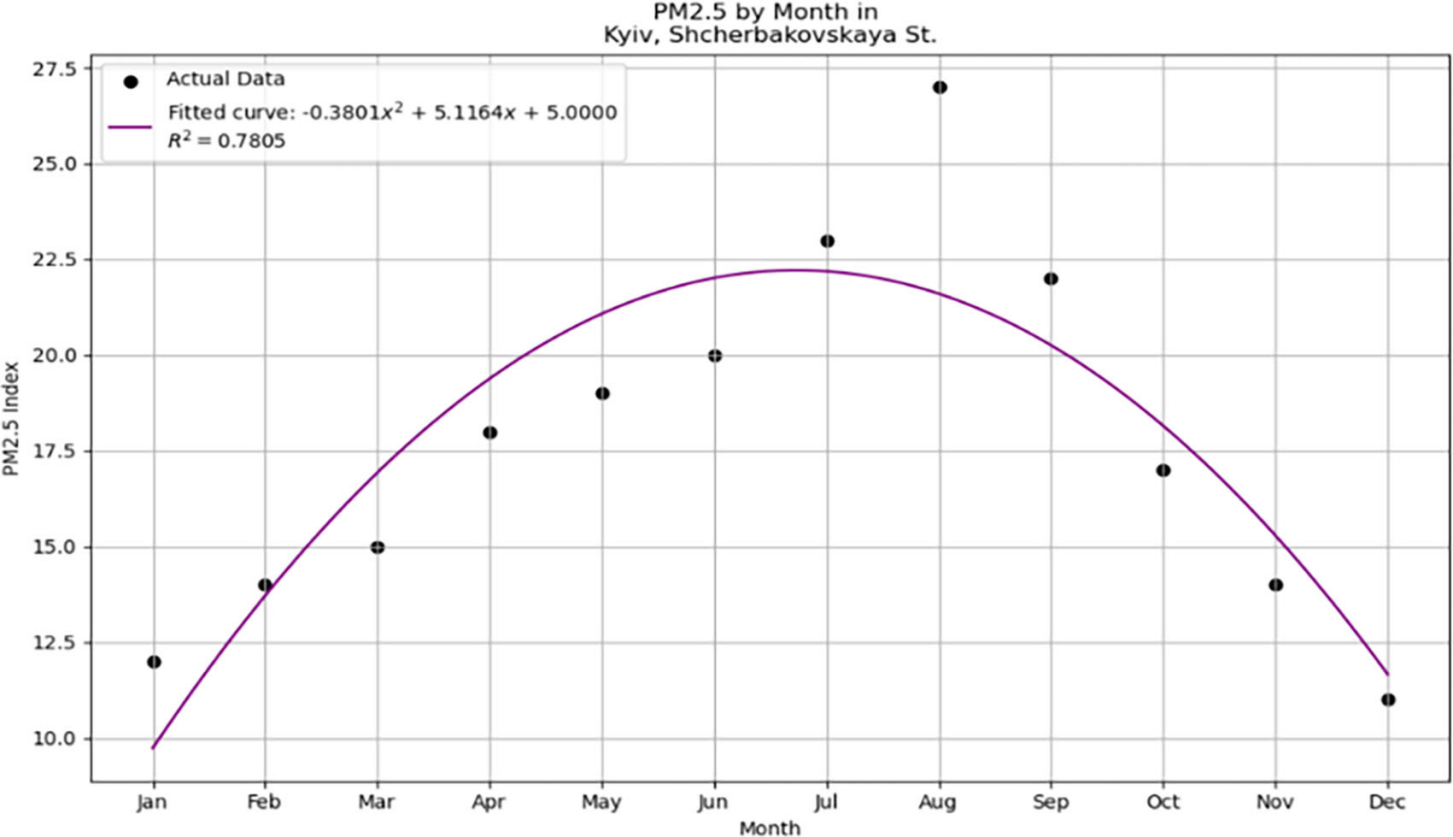
Quadratic polynomial fit of dataset.

The quadratic polynomial fitting method used in this study demonstrates Python's ability to effectively manage and analyze complex datasets. The datasets used herein are illustratively generated, serving as a basis for demonstrating the potential applications of quadratic polynomial models. The fitting process provided a smooth curve aligned closely with the observed data points, indicating robust model performance. Notably, the computed coefficient of determination, R-squared $\left(R^{²}\right),$ was substantially high, reflecting a strong correlation between the observed values and those predicted by the model. This statistical measure underpins the polynomial's ability to capture and explain variability in the data effectively, which is crucial for validating the regression model used in this analysis. Figure 1 illustrates the quadratic polynomial curve fitted to the observed data points using Python's plotting library Matplotlib.

The curve represents the model obtained from regression analysis, where the quadratic polynomial provides a significant fit to the data, as evidenced by the computed R-squared value. The axes were labeled to identify the independent variable (x-axis) and dependent variable (y-axis), and a legend was included to differentiate between the observed data points and fitted polynomial curve. The smoothness of the curve indicates the effectiveness of the model in capturing trends within the dataset, which can be utilized for predictive analytics and further statistical inferences. After configuring the plot with the necessary parameters for clear and informative visualization, it was generated using the 'plt. show()' function in Matplotlib.

## 4. Discussion

Quadratic polynomials are valued for their ability to model nonlinear relationships in various data contexts, balancing computational efficiency, and interpretability. However, their performance can be limited when confronted with complex multivariable systems in which more sophisticated statistical models may be more accurate. Future research could address these challenges by focusing on several advancements in quadratic polynomial modeling. Incorporating regularization techniques is recommended to counteract overfitting, particularly for datasets with intricate structures. Exploring hybrid models that merge the clear interpretive benefits of quadratic polynomials with the robust capabilities of machine learning algorithms could also enhance predictive accuracy.

Moreover, the development of adaptive polynomial models that adjust their parameters based on real-time data inputs can significantly improve the dynamic data analysis. Extending these models to operate within multiscale frameworks may offer deeper insights into various levels of data structure, ensuring a comprehensive understanding of complex patterns. These enhancements are crucial for extending the utility of quadratic polynomials beyond their current capabilities and facilitating more accurate and efficient statistical analyses across diverse datasets.

This study acknowledges that the effectiveness of quadratic polynomials, like any statistical model, is contingent on the quality and volume of the data available. To mitigate potential biases and inaccuracies in the input data, the data collection methodology should include rigorous data preprocessing steps, such as outlier removal, normalization, and feature selection, which are crucial for enhancing the reliability of the research. Despite the potential of more advanced models, this study primarily advocates quadratic polynomials because of their suitability for datasets exhibiting quadratic relationships, which are frequently encountered in environment-related target research. However, future research should continue to explore the comparative dynamic performance of quadratic polynomials, for example, the performance of benchmarking against contemporary machine-learning algorithms to ensure a comprehensive understanding of their relative merits, possibly extending the use of hybrid approaches that combine the strengths of traditional polynomial models and cutting-edge machine-learning techniques.

While quadratic models offer simplicity and clarity, they may only capture part of the complexity of data as effectively as some machine-learning models. However, their computational efficiency and suitability for smaller datasets can be advantageous for specific scenarios.

## 5. Conclusion

These findings highlight the practical utility of the quadratic polynomials in Python for predictive analytics. The application of these polynomials in regression analysis, as demonstrated through Python scripts and methodologies, bridges theoretical concepts with real-world data analytics and enhances the interpretative power of statistical models in research. The high R-squared value obtained confirms the model's accuracy and predictive performance, making it a valuable tool for researchers and analysts seeking to conduct sophisticated data analyses. It is important to acknowledge, however, that the dataset used in this study were illustratively generated, which may limit the generalizability of the results to real-world datasets with more complex and unpredictable patterns. Furthermore, integrating Python libraries such as NumPy and Matplotlib in this process underscores the adaptability and efficiency of Python for handling complex and nuanced datasets across various research domains.

## Ethical compliance

All procedures involving human participants were performed in accordance with the ethical standards of the Institutional and National Research Committee.

## Author contributions

Rostyslav Sipakov contributed to the research design, implementation, and manuscript writing. Dr. Voloshkina and Dr. Kovalova helped implement and analyze the results. All authors have seen and agreed to the final content of the manuscript.

## Data Availability

No data is associated with this article.

## Software availability statement

• Source code of the scripts available from: https://github.com/rsipakov/QuadraticPolynomialsPyDA
• Archived scripts available from: https://doi.org/10.5281/zenodo.10637508
• License: OSI approved open license software is under MIT License ( https://opensource.org/license/MIT)
