# Supplementary material for: Leveraging Quadratic Polynomials in Python for Advanced Data Analysis
Source: F1000Res. 2024 Aug 20;13:490. Originally published 2024 May 17. [Version 2] doi: 10.12688/f1000research.149391.2 (PMC11375405; doi:10.12688/f1000research.149391.2)
Supplement: Peer review history

# Reviewer report: Reviewer response for version 2

**DOI:** 10.5256/f1000research.169942.r315474

**Published:** 2024-09-04

**Authors:** Qiao Wang

**Affiliations:** 1 School of Economics and Management, Southeast University, Nanjing, China; 2 Southeast University, Nanjing,, China

The quadratic polynomial is a fundamental modeling technique in data analysis, particularly because any smooth function can be approximated by a quadratic polynomial at a fixed point using a second-order Taylor expansion. This makes it a valuable concept for experts and researchers in the medical field to understand. 

 This revised version partially addressed my concerns in my initial review. However, it still avoids the more usual contents concerning multivariate model.

Are the conclusions about the tool and its performance adequately supported by the findings presented in the article?

Partly

Is the rationale for developing the new software tool clearly explained?

No

Is the description of the software tool technically sound?

No

Are sufficient details of the code, methods and analysis (if applicable) provided to allow replication of the software development and its use by others?

No

Is sufficient information provided to allow interpretation of the expected output datasets and any results generated using the tool?

Partly

Reviewer Expertise:

Data analytics

I confirm that I have read this submission and believe that I have an appropriate level of expertise to confirm that it is of an acceptable scientific standard.

# Reviewer report: Reviewer response for version 2

**DOI:** 10.5256/f1000research.169942.r315475

**Published:** 2024-09-03

**Authors:** Selim Molla

**Affiliations:** 1 The University of Texas at El Paso, El Paso, Texas, USA

**Overall Assessment:** The article *"Leveraging Quadratic Polynomials in Python for Advanced Data Analysis"* is well-constructed and provides a valuable tool for researchers seeking to apply quadratic polynomials using Python. The authors have successfully demonstrated the tool's capability to model nonlinear relationships in data, offering practical guidance and sufficient details for replication. The work is technically sound and offers a clear rationale for the development of the software tool.

**Recommendation for Multivariable Analysis:** While the article is a strong contribution, I recommend that the authors consider expanding the scope to include multivariable analysis. The current focus on single-variable quadratic polynomials limits the applicability of the tool in more complex, real-world scenarios where multivariable models are often required. By incorporating or discussing multivariable quadratic polynomial models, the article would significantly enhance its relevance and utility for a broader audience.

**Conclusion:** I approve the article for indexing but strongly recommend including multivariable analysis in the current version or future updates. This addition would greatly improve the comprehensiveness and applicability of the tool, making it even more valuable for the research community.

Are the conclusions about the tool and its performance adequately supported by the findings presented in the article?

Yes

Is the rationale for developing the new software tool clearly explained?

Yes

Is the description of the software tool technically sound?

Yes

Are sufficient details of the code, methods and analysis (if applicable) provided to allow replication of the software development and its use by others?

Yes

Is sufficient information provided to allow interpretation of the expected output datasets and any results generated using the tool?

Yes

Reviewer Expertise:

Mathematical modeling, Discrete computer modeling and simulation, Machine learning, and Data analytics

I confirm that I have read this submission and believe that I have an appropriate level of expertise to confirm that it is of an acceptable scientific standard.

# Reviewer report: Reviewer response for version 1

**DOI:** 10.5256/f1000research.163848.r290970

**Published:** 2024-08-08

**Authors:** Qiao Wang

**Affiliations:** 1 School of Economics and Management, Southeast University, Nanjing, China; 2 Southeast University, Nanjing,, China

This paper explores modelling using quadratic polynomials in Python for data analysis applications. However, the study is limited to the single variable case, which does not meet the standards of current applications. In regression analysis, multivariate quadratic polynomial models or more generalized higher order polynomial regressions are typically used, but these crucial aspects are not covered in the current work.  I have comments as below:

 1. The "methods"  outlined in this article lack distinction, since it is a naive description on tool software; as a research article, they should be more prominently highlighted.

 2. The quadratic polynomial discussed in Section 2.1 mainly addresses the single-variable case. However, in a broader viewpoint, polynomial regression encompasses multivariable contexts, with its theoretical underpinnings and algorithms applied across various fields. I believe the explanation in this section could be revised and enhanced extensively.

 3. This entire subsection 2.1 could be reformulated using quadratic forms, along with pertinent linear algebra and matrix theory. Failing to do so would limit the model to a single-variable scenario.

 4. Section 3 solely demonstrates a standard example in the single-variable scenario. However, I recommend improving the clarity of its capabilities and limitations by introducing a counterexample, such as a dataset that fits well with a cubic curve but may fail when approximated with quadratic polynomial models.

 To summarize, this article's discussion on the Python library for quadratic regression modeling is limited to single-variable scenarios and does not effectively showcase the model's fitting capabilities. In my opinion, the content is overly simplistic and does not adequately address practical data analysis applications. Therefore, a thorough major revision is necessary.

Are the conclusions about the tool and its performance adequately supported by the findings presented in the article?

Partly

Is the rationale for developing the new software tool clearly explained?

No

Is the description of the software tool technically sound?

No

Are sufficient details of the code, methods and analysis (if applicable) provided to allow replication of the software development and its use by others?

No

Is sufficient information provided to allow interpretation of the expected output datasets and any results generated using the tool?

Partly

Reviewer Expertise:

Data analytics

I confirm that I have read this submission and believe that I have an appropriate level of expertise to confirm that it is of an acceptable scientific standard, however I have significant reservations, as outlined above.

# Response

**Published:** 2024-08-12

**Authors:** Rostyslav Sipakov

**Affiliations:** Environmental Protection, Kyiv National University of Construction and Architecture, Kyiv, Ukraine

Dear Dr. Wang,

 Thank you very much for your review, which is of significant importance to our study.

 Although we aimed to present a simple and accessible tool for researchers with basic knowledge of Python programming, the comments from the previous reviewer regarding pre-processing and limitations and your comments regarding multivariate quadratic polynomial models will allow us to present a substantial version of this tool.

 For technical reasons, your comments were published after we submitted a new version (revision 1) of the manuscript to the editorial office based on the previous reviewer's comments. Nevertheless, we have begun work on a new version (revision 2) of the manuscript to reflect your valuable comments.

 Thank you once again for providing the opportunity to significantly improve our study.

 Sincerely,

 Dr. Sipakov

# Reviewer report: Reviewer response for version 1

**DOI:** 10.5256/f1000research.163848.r305465

**Published:** 2024-07-29

**Authors:** Selim Molla

**Affiliations:** 1 The University of Texas at El Paso, El Paso, Texas, USA

The article is fundamentally sound and makes a significant contribution to the application of quadratic polynomials in data analysis using Python. However, to enhance clarity, completeness, and practical utility, the following revisions are recommended:

**Introduction: Detailed Explanation of Suitability**

**Improvement:** Provide a more detailed explanation of why quadratic polynomials are particularly suitable for modelling and analysing data, compared to other polynomial or non-linear models.

**Details:** Explain the unique advantages of quadratic polynomials in capturing curvature and non-linear relationships. Compare briefly with other models (e.g., linear, cubic polynomials) to highlight the specific scenarios where quadratic polynomials are most effective.

**Methods: Data Preprocessing Steps**

**Improvement:** Include a more detailed explanation of the data preprocessing steps, such as handling missing values or outliers.

**Details:** Add a subsection detailing the preprocessing steps taken before fitting the quadratic model. This should include techniques for handling missing data, outlier detection and treatment, and any data normalization or scaling applied.

**Methods: Discussion of R-squared Limitations**

**Improvement:** Briefly discuss the limitations of using R-squared as the sole measure of model fit and suggest additional metrics.

**Details:** Include a paragraph explaining that while R-squared is useful, it has limitations, especially in non-linear contexts. Suggest other metrics like Adjusted R-squared, Mean Squared Error (MSE), or Root Mean Squared Error (RMSE) to provide a more comprehensive evaluation.

**Results: Comparison with Other Models**

**Improvement:** Include a comparison with other models, such as linear regression or higher-degree polynomials, to provide a broader perspective on the performance of quadratic models.

**Details:** Add a section that compares the performance of quadratic polynomials with linear and cubic models using the same datasets. Use metrics like R-squared, Adjusted R-squared, and MSE to compare the fit and predictive accuracy.

**Discussion: Challenges and Limitations**

**Improvement:** Delve deeper into the potential challenges and limitations of quadratic polynomials, such as overfitting or sensitivity to data variability.

**Details:** Discuss scenarios where quadratic polynomials might overfit, particularly with small or noisy datasets. Provide suggestions for mitigating these issues, such as regularization techniques or cross-validation methods. Mention the sensitivity of quadratic models to data variability and how to handle such cases.

Are the conclusions about the tool and its performance adequately supported by the findings presented in the article?

Yes

Is the rationale for developing the new software tool clearly explained?

Yes

Is the description of the software tool technically sound?

Yes

Are sufficient details of the code, methods and analysis (if applicable) provided to allow replication of the software development and its use by others?

Yes

Is sufficient information provided to allow interpretation of the expected output datasets and any results generated using the tool?

Yes

Reviewer Expertise:

Mathematical modeling, Discrete computer modeling and simulation, Machine learning, and Data analytics

I confirm that I have read this submission and believe that I have an appropriate level of expertise to confirm that it is of an acceptable scientific standard, however I have significant reservations, as outlined above.

# Response

**Published:** 2024-08-01

**Authors:** Rostyslav Sipakov

**Affiliations:** Environmental Protection, Kyiv National University of Construction and Architecture, Kyiv, Ukraine

Dear Selim Molla,

 Thank you very much for your valuable comments. Based on your comments, we have revised and highlighted the article in red font. Due to the significant volume of changes we have made, including new figures, we provide a brief description of what we have done below.

 The full text of the preprint of revision 1 can be obtained via the following link: https://arxiv.org/abs/2402.06133.

 Please wait for the updated version (version 3) to be published.

 Announcement Schedule Thursday 20:00 (08/01/2024)

 Also, a new version has been submitted to the editorial office, and we hope it will be published soon. Within one business day, we will also publish the updated code on GitHub.

**What has been done:**

**Point 1.** Introduction: Detailed Explanation of Suitability.

**Response 1:** This paper highlights quadratic polynomials' unique advantages in capturing curvature and non-linear relationships. It also includes a brief comparison with other models, such as linear and cubic polynomials, emphasizing specific scenarios where quadratic polynomials are most effective.

**Point 2:** Methods: Data Preprocessing Steps

**Response 2: **A new subsection has been added detailing the preprocessing steps undertaken before fitting the quadratic model. This includes techniques for handling missing data, detecting and treating outliers, and applying data normalization or scaling. This addition aims to provide a clearer understanding of the steps taken to prepare the data for analysis.

**Point 3: **Methods: Discussion of R-squared Limitations

**Response 3: **The methods section now contains a paragraph discussing the limitations of using R-squared as the sole measure of model fit. It suggests additional metrics such as Adjusted R-squared, Mean Squared Error (MSE), and Root Mean Squared Error (RMSE) to offer a more comprehensive evaluation of model performance.

**Point 4:** Results: Comparison with Other Models

**Response 4: **A new section has been included comparing the performance of quadratic polynomials with linear and cubic models using the same datasets. The comparison uses metrics like R-squared, Adjusted R-squared, and MSE to evaluate the fit and predictive accuracy, providing a broader perspective on the effectiveness of quadratic models.

**Point 5:** Discussion: Challenges and Limitations

**Response 5: **The discussion now delves deeper into the potential challenges and limitations of quadratic polynomials, such as overfitting and sensitivity to data variability. It addresses scenarios where quadratic polynomials might overfit, particularly with small or noisy datasets, and suggests methods for mitigating these issues, including regularization techniques and cross-validation methods. This addition aims to provide a more balanced view of the use of quadratic polynomials in data analysis.

# Response

**Published:** 2024-08-20

**Authors:** Rostyslav Sipakov

**Affiliations:** Environmental Protection, Kyiv National University of Construction and Architecture, Kyiv, Ukraine

Dear Selim Molla,

 We have received a notification from the editorial board that version 2 of our manuscript has been publicly published. However, we are currently working on version 3 to address the comments provided by another reviewer. Nonetheless, we would greatly appreciate your feedback regarding your significant conceptual remarks, which we have endeavored to incorporate into version 2 of our manuscript. Once again, thank you very much for your attention and for providing valuable conceptual insights into our work.

 Sincerely,

 Dr. Sipakov
